# Investment in Cancer Prevention and Care for Forcibly Displaced Syrians Is an Urgent Priority

**DOI:** 10.1200/GO.22.00382

**Published:** 2023-01-03

**Authors:** John W. Carew, Mohamed Hamze, Bassel Atassi, Aula Abbara, Kaveh Khoshnood

**Affiliations:** ^1^ Science Health Education Center, Dana Farber Cancer Institute, Boston, MA; ^2^ Syrian American Medical Society, Washington, DC; ^3^ OSF Little Company of Mary Medical Center, Evergreen Park, IL; ^4^ Department of Infectious Diseases, Imperial College, London, United Kingdom; ^5^ Yale School of Public Health, Yale University, New Haven, CT

## Introduction

The international community is failing forcibly displaced people in cancer prevention and care. Despite the significant burden that cancer and other noncommunicable diseases (NCDs) weigh on internally displaced people (IDPs) and refugee populations, international organizations have been unable to devote adequate resources to address these issues and have historically concentrated on communicable diseases prevention among the forcibly displaced.^1^ The Arab spring and related conflicts and forced displacement have catalyzed discussions in the humanitarian sector about the needs for prevention, diagnosis, and management of cancer and other NCDs. However, there remain challenges related to underresourcing, deprioritization of chronic diseases (particularly those without overt or immediate symptoms), difficulties following up transient populations, and in some instances a lack of expertize.^2^ Despite efforts to invest in primary health care for displaced populations, significant underresourcing continues to prevent the development of comprehensive strategies targeting cancer and other NCDs.^3-5^ The lack of resources, including funding, expertize, diagnostics, and therapeutics, negatively effects the three main domains of cancer mortality prevention: primary prevention, early diagnosis and screening, and cancer management.

Women and children who are forcibly displaced may face the burden of health concerns which not only include NCDs but also sexual and reproductive health needs and psychosocial trauma. They may also face challenges around cancer diagnosis and care, particularly breast and cervical cancer which pose especially severe burdens because of lack of access to screening mammography services, unattainably high costs of care, and stigma.^6,7^ Such factors do not only affect forcibly displacement women in transit but also in host or resettlement countries. For example, rates of advanced stage breast and cervical cancer have been shown to be higher among refugee and immigrant women than women born in the United States or Canada.^6^

Since uprisings Syrian began March 2011 escalated into violent conflict, more than half the prewar population of 22 million have been forcibly displaced: 6.7 million as IDPs and 15.3 million as refugees, most of whom are in countries neighboring Syria.^8^ Conditions for Syrian IDPs and refugees are stark with particularly challenges around accessing health care, particularly for expensive or complex care which includes cancer care.^8^

## Disproportionately High Rates of Carcinogen Exposure Among Forcibly Displaced Syrians

Forcibly displaced Syrians in Syria and those who are refugees face a high exposure to carcinogens and structural risk factors putting them at risk of high cancer rates.^9^ Syrian refugees in particular experience high rates of exposure to carcinogens, including those leading to breast and cervical cancers.^10^ Perniciously, these risk factors are largely outside of their control. This is particularly so for environmental exposures for refugees in vulnerable or precarious settings such as Lebanon, where many refugees reside in tented settlements. A recent Human Rights Watch report found that burning landfills and trash heaps are often situated near marginalized refugee communities.^11^ Artisanal oil refineries also operate near these communities, which spread carcinogens via air and groundwater, when floods distribute contaminated waste wells to drinking water sources.^12^ Throughout the winter, individuals and families living in substandard housing must burn rubbish and plastic to survive bitterly cold temperatures, creating dangerous indoor air pollution.^13^ Overall, these environmental sources of air and water pollution may be responsible for apparently high rates of cancer among refugees.

Modifiable behaviors may also contribute to cancer incidence among forcibly displaced Syrians. Syrian refugees exhibit high smoking rates, especially among males, of whom more than 30% smoke; smoking is associated with an increased risk of lung and bronchus, bladder, and laryngeal cancer.^14-16^ In preconflict Syria, public health measures were starting to be introduced with regulations around smoking; however, no such public health measures exist for IDPs or refugees after the uprisings. Smoking cessation is challenging even outside of such desperate settings; however, among Syrian IDPs and refugees, various social, cultural, and trauma-related factors need to be considered. It is suggested that in some cases, smoking rates may have reduced because of the extreme poverty forcibly displaced Syrians face while for others, rates may have increased including among women where previous gendered social norms may have been challenges.

The targeting of preventative public health measures for cancer toward the specific needs of Syrian IDPs and refugees in various contexts is essential to understanding the drivers. This needs to take a multipronged approach at the policy level, including the removal or reduction of environmental carcinogens, risk factor reduction, and potentially for targeted low cost screening interventions. Although the humanitarian response for IDPs and refugees is vastly underfunded, there may be potential screening programs which may be economically viable in the longer term.

Tailored approaches are key as even where national initiatives to promote healthy behaviors do exist, they oftentimes fail to specifically target refugee and displaced communities and are linguistically, geographically, or culturally inaccessible.^17^ Thus, these campaigns may lead to increased cancer prevention among nationals of a country but have no or only partial impact on especially at-risk communities.^17^

## Missed Cancer Diagnoses Among Forcibly Displaced Syrians

Furthermore, although countries hosting large numbers of refugees and displaced persons have made significant progress in setting up cancer screening and early detection programs, refugees often cannot access these services either because of a lack of inclusive health policies or addressing structural factors which discriminate against refugees in these countries.^17^ Lebanon and Turkey, two countries that host the largest per capita and absolute numbers of refugees, respectively, in the Middle East and North Africa region, lack widely used cancer screening and surveillance programs.^18^ Studies report low awareness and usage of cancer screening among Syrian refugees in Turkey.^19^ Furthermore, low reported cancer rates among Syrian refugees in Jordan compared with host populations indicate pervasive underdiagnosis and underreporting.^20^

Even after diagnosis, accessing cancer treatment for IDPs or Syrian refugees can be out of reach. The provision of often expensive services which may not be prioritized alongside immediately life-threatening conditions which IDPs or refugees suffer with is often underresourced and heavily scrutinized. Oftentimes, refugees must make the case to international agencies that their prognosis is good enough to be worth treating, even when funding and services are available.^21^ Even after surmounting the complicated bureaucratic barriers, between 2010 and 2012, only 48% of the 511 applications by refugees for cancer treatment in Jordan were approved.^9^ For IDPs, cancer treatment is often out of reach because of geographical location, political barriers eg cross-line, a lack of availability of specialists or treatments, and the extremely high cost in a country where more than 90% live in poverty.^17^

## Cancer Prevention as a Key Intervention for Refugees

Primary cancer prevention has the potential to prevent cancer and subsequent pressures on humanitarian responses and health systems. However, key aspects of cancer prevention, such as behavior change, are stymied or made entirely impossible by the legal and political restrictions placed on displaced persons and refugees. Strict restrictions on movement and extremely limited access to healthy foods can make it impossible for these vulnerable communities to take steps to lower their personal cancer risk.^22^ For example, refugees living in overcrowded camps may spend significant amounts of their days queuing in lines to address administrative issues, receive food rations or stipends, access water, sanitation, and hygiene, or access health care.^23^ Additionally, they may hold unresolved past or current traumas which affect motivation for behavior change.^24^

Thus, leaders and decision makers must enact decisions that build healthy environments to lower exposure to risk factors and promote healthy behaviors on a community level before behavior change interventions can begin to lower rates of cancer.^25,26^ Such policies, like mandatory human papillomavirus vaccination and smoke-free buildings and neighborhoods, can effectively lower rates of cancer.^27^ Although globally cancer prevention campaigns have already had success achieving individual- and community-level cancer prevention, there is much room for growth in breast and cervical cancer prevention campaigns; these may include simple measures such as education on self-checking of breasts for lumps either opportunistically at clinic visits or in a more structured way.^28^ Efforts to vaccinate refugee women and girls against human papillomavirus and provide healthy food to refugee camps and other vulnerable settings have the potential to produce significant benefits.

Although behavioral interventions are one piece of the puzzle, efforts are also urgently needed in the policy arena to improve the environments of forcibly displaced communities. Programs to limit air pollution have the potential to significantly contribute to cancer prevention, especially considering the aforementioned poor air quality resulting from burning rubbish and rubber and artisanal oil refineries near refugee camps and other forcibly displaced communities.^29^

In addition to reducing mortality from breast and cervical cancer, preventing cases of cancer allows individuals to avoid stigmatization and other deleterious social effects that accompany a cancer diagnosis in many parts of the world.^30^ Preventing cancer is also an extremely cost-effective opportunity to decrease costs and prevent future mortality and morbidity. Increased investment in upstream factors that influence cancer risk will pay off in the long run. Organizations working with refugee and displaced communities should increase their investments in cancer awareness and prevention activities to promote better health outcomes and reach larger populations. Cancer prevention efforts will decrease cancer rates, which will in turn allow organizations to better spend funds on cancer care.

## Cancer Care for IDPs in Syria

Before the uprising, cancer care in Syria was heavily centralized with the main centers and specialists concentrated in Damascus and Aleppo cities.^9^ This has affected cancer care currently, particularly in the northwest which remains outside of government control and the northeast which is under the autonomous administration of northeast Syria.^31^ Given the gaps in cancer care for IDPs and host populations in northwest Syria as well increasing difficulties of crossing to Turkey for treatment (particularly during the COVID-19 pandemic), humanitarian organizations have needed to step in to provide high-cost interventions such as oncology care.^31^ The Syrian American Medical Society (SAMS) hassupported histopathology diagnosis and three oncology treatment centers in northwest Syria.^32^ These centers provide a step-by-step comprehensive oncology program to the most vulnerable people in Syria, in a region that shelters 4.2 million, of whom 58% are IDPs.^33^ SAMS centers provide imaging, diagnostic tests, surgery, chemotherapy, and follow-up psychological support.^34^ Patients with suspected or confirmed cancer in northeast Syria have limited access to needed care with limited options, one of which is to go areas under government control which is not an option for many in the area.^35^

In conclusion, despite the challenges posed by responding to cancer and other NCDs in Syria and other Eastern Mediterranean countries, organizations such as SAMS, the WHO, and the United Nations High Commissioner for Refugees have risen to the task of addressing these costly and complicated health issues including with innovations.^3,32^ Given the worse prognosis and higher costs associated with late diagnosis of cancer, prevention is an essential consideration for IDPs and refugees. Promoting healthy behaviors now could reduce cancer mortality through earlier diagnosis, financial burdens, social stigmatization, and poor quality of life down the road, reducing the burden on the individual and on the health system. Forcibly displaced women who may be at particularly at risk for breast and cervical cancer mortality because of diagnostics delays^36^ stand to benefit the most from a renewed focus on and funding for cancer prevention. Thus, it is imperative that international and local organizations serving these women implement new cancer prevention programs appropriate to their contexts. Enormous strides have been made in lowering communicable disease-related mortality among forcibly displaced people, and we must not let NCDs or cancer hamper such progress. An increased focus on cancer and NCDs in settings of forced displacement or other humanitarian contexts will contribute to ensuring the United Nations–recognized principle that health is a human right.
